# Diagnosis of Isolated Saccular Dysfunction Using Trapezius cVEMP: A Detailed Vestibular Assessment

**DOI:** 10.3390/diagnostics15232988

**Published:** 2025-11-25

**Authors:** Mădălina Georgescu, Oana Irina Popa, Horațiu Ștefănescu, Violeta Necula, Alma Maniu, Irina Enache, Andrei Osman

**Affiliations:** 1 Department of General Surgery and Qualified Care in Surgical Specialties, “Carol Davila” University of Medicine and Pharmacy, 050474 Bucharest, Romania; madalina.georgescu@gecad.com; 2 Otorhinolaryngology Department, “Dr. Carol Davila” Central Military Emergency University Hospital, 010825 Bucharest, Romania; oana.irina@gmail.com; 3 Department of Otolaryngology, “Victor Babeş” University of Medicine and Pharmacy, 300041 Timişoara, Romania; 4 Department of Otorhinolaryngology, “Iuliu Hatieganu” University of Medicine and Pharmacy Cluj-Napoca, 400012 Cluj-Napoca, Romania; violeta.necula@elearn.umfcluj.ro (V.N.); aurelia.maniu@umfcluj.ro (A.M.); 5 Department of Anatomy and Embriology, University of Medicine and Pharmacy of Craiova, 200349 Craiova, Romania; irina.enache@umfcv.ro (I.E.); andrei.osman@umfcv.ro (A.O.)

**Keywords:** imbalance, dizziness, saccular dysfunction, vestibular syndrome, trapezius, vestibular evoked myogenic potentials, cVEMP, nystagmus, videonystagmography, posturography

## Abstract

**Background and Clinical Significance**: Vestibular disorders include a wide range of conditions with overlapping symptoms such as dizziness, vertigo and imbalance, often offering diagnostic challenges when distinguishing between peripheral and central etiology. Accurate differentiation is essential for establishing effective treatment plans. In rare or atypical cases with subtle findings, comprehensive diagnostic tools—such as extended vestibular tests and structured questionnaires like the Dizziness Handicap Inventory (DHI)—are critical for diagnosis and monitoring patient recovery. **Case Presentation**: A 35-year-old female presented with chronic imbalance and motion-induced dizziness persisting for four years. The patient had a surgical history of right-sided functional neck dissection for a parotid tumor. A comprehensive audiovestibular evaluation was performed, including pure tone audiometry (PTA), tympanometry, videonystagmography (VNG), cervical vestibular evoked myogenic potentials (cVEMP), ocular vestibular evoked myogenic potentials (oVEMP), video head impulse testing (vHIT), computerized dynamic posturography (CDP), and magnetic resonance imaging (MRI). The Dizziness Handicap Index (DHI) was administered at baseline and post-treatment to monitor subjective symptom changes. Objective testing revealed marked right–left amplitude asymmetry on cVEMP, which were recorded from the trapezius muscle due to prior neck dissection surgery, indicating isolated right-sided saccular hypofunction. Following targeted vestibular rehabilitation and pharmacologic treatment, the 3-month reassessment demonstrated resolution of symptoms and a reduction in DHI scores from 24 to 6. **Conclusions**: Comprehensive vestibular testing, performed in a single diagnostic session, enabled the accurate identification of isolated right-sided saccular hypofunction in this complex post-surgical case. Combining cVEMP, CDP, and DHI assessment provided a complete functional profile, guided targeted rehabilitation, and allowed objective monitoring of recovery.

## 1. Introduction

Instability during walking, dizziness or even vertigo provoked by moving one’s head can be indicative of either inner ear problems or central neurological issues. Symptoms like these are often associated with dysfunctions in the vestibular system, which includes both peripheral analyzers in the inner ear and central components in the brain [1,2]. While inner ear problems are a frequent cause of dizziness or vertigo, central neurological issues can also manifest similar symptoms [3]. Differentiating between these etiologies (peripheral or central) is essential for effective patient management [4].

Vestibular testing is a crucial for step for diagnosing and differentiating peripheral disorders from central lesions, and tests like cVEMP, oVEMP, and video head impulse testing (vHIT) play significant roles in this domain [5], alongside physical tests and hearing assessment [6]. These tests provide comprehensive insights into the functioning of the otolithic organs and semicircular canals, which are essential for balance and spatial orientation. The integration of these tests into vestibular evaluation enhances the diagnostic accuracy for inner ear disorders. cVEMP and oVEMP are used to assess the saccule and utricle, respectively, along with their associated vestibular nerve pathways. cVEMP evaluates the saccule and the inferior vestibular nerve, while oVEMP assesses the utricle and the superior vestibular nerve [7,8]. These tests are useful in diagnosing peripheral lesions like Meniere’s disease (MD), benign paroxysmal positional vertigo (BPPV) and superior semicircular canal dehiscence (SSCD). For instance, cVEMP can detect endolymphatic hydrops in MD and abnormal communications between middle and inner ear like in SSCD, while oVEMP abnormalities are common in BPPV patients [9,10,11]. In rare occurrences, vHIT combined with cVEMP and oVEMP may lead to diagnosing isolated saccular or utricular dysfunctions [12].

vHIT is used to assess the function of the semicircular canals, horizontal and vertical alike. It is crucial for diagnosing and monitoring disorders like vestibular neuritis and bilateral vestibulopathy, its clinical and research uses extending beyond these clinical entities [13]. vHIT is particularly useful in identifying isolated dysfunctions of the semicircular canals, such as posterior semicircular canal dysfunction, which might not be detected by other vestibular tests [13]. When used alongside cVEMP and oVEMP, vHIT provides a complete assessment of the vestibular labyrinth, allowing for a more detailed understanding of vestibular disorders and their impact on balance and spatial orientation [14].

In order to perform a good assessment of the inner ear, and provide further information for differential diagnosis, hearing tests, particularly PTA, are frequently performed. They serve a critical role in diagnosing and managing vestibular diseases such as MD and sudden sensorineural hearing loss (SSNHL). Hearing tests help in assessing the extent of inner ear involvement. For MD, for example, PTA allows differentiation differentiating between stages of the disease and even predicting prognosis. PTA, which measures hearing sensitivity across different frequencies, is often used in conjunction with vestibular function tests to provide a comprehensive evaluation of the inner ear. PTA is a crucial test for diagnosing MD, as it helps identify characteristic patterns of hearing loss, such as low-frequency sensorineural hearing loss, which is typical in early stages of the disease [15,16]. In SSNHL, PTA help classify the severity and frequency configuration of the hearing loss, which correlates with vestibular dysfunction and prognosis. For instance, severe and profound sensorineural hearing loss often indicates more extensive vestibular involvement [17,18]. For SSNHL patients, PTA is also the primary test used to monitor hearing recovery [17].

Additional tests can be used to further assess vestibular function and associated disorders. Posturography plays a significant role in the diagnosis and management of vestibular diseases, offering a comprehensive evaluation of balance control by integrating sensory inputs from the vestibular, visual, and somatosensory systems. It is particularly valuable in differentiating peripheral vestibular disorders from other conditions, which is crucial for selecting appropriate treatment strategies. CDP provides an objective measure of a patient’s ability to use vestibular information, making it an essential tool in both diagnosis and rehabilitation of vestibular disorders [19,20]. Studies have shown that CDP, especially when combined with head movements, improves the sensitivity and specificity of diagnosing vestibular impairments. This is particularly evident in conditions where traditional tests may not fully be able to evaluate the extent of vestibular dysfunction [20].

MRI is a critical tool in diagnosing unclear vestibular diseases, offering high diagnostic accuracy for certain conditions. MRI is particularly effective in identifying vestibular schwannomas, with sensitivity and specificity close to 100% [21]. While MRI is invaluable for diagnosing certain vestibular disorders, its effectiveness can be limited by the specific disorder and the timing of the imaging. For instance, in hyperacute central vestibular syndromes, MRI may not detect small strokes immediately, necessitating a combination of clinical assessment and imaging for accurate diagnosis [6,22]. Additionally, the differentiation between certain diseases like vestibular migraine and MD remains challenging, as both conditions share overlapping symptoms, and no single test is sufficiently specific on its own [23], despite advanced imagining techniques.

Despite the large battery of tests and investigations, patients’ symptoms are not always highlighted by abnormalities measurable by tests or by MRI images. Several questionnaires are available to help monitor symptom severity and patient recovery. DHI is a frequently used tool for assessing the recovery of vestibular patients, as it provides a comprehensive evaluation of the impact of dizziness on a patient’s life [24]. The DHI is widely used in clinical settings to quantify the severity of dizziness-related disability and to monitor changes over time, making it an essential component of vestibular rehabilitation and treatment evaluation [25,26].

## 2. Detailed Case Presentation

This case report is based on clinical data collected during the patient’s initial admission to the Central Military Emergency University Hospital in Bucharest, Romania, as well as results from the follow-up evaluation at three months after completing the treatment and customized vestibular rehabilitation program. All presented results were obtained from the patient’s hospital examination files, after receiving written consent from the patient. Clinical data were processed and summarized using Microsoft Office Professional Plus 2019 (Microsoft Corporation, Redmond, WA, USA) for documentation, graphical presentation and text integration.

**Figure 1:**
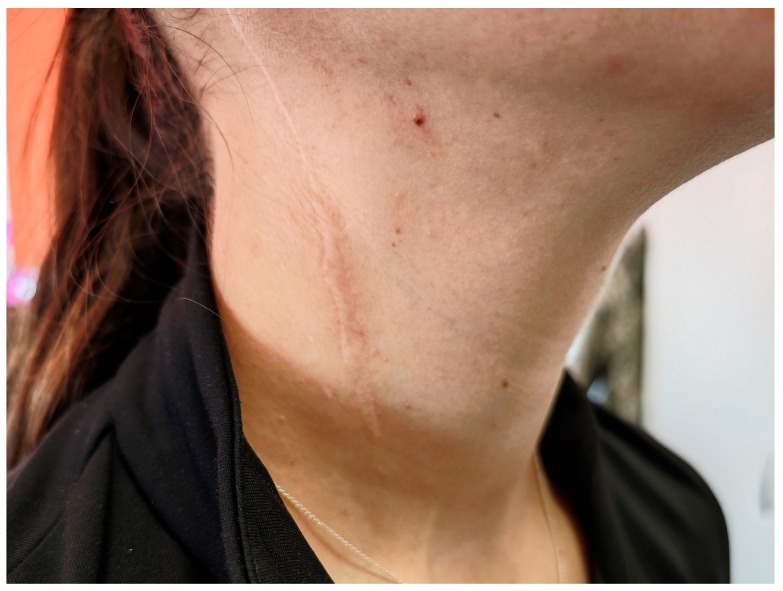
Right-sided cervical scar, post-functional neck dissection.

We report the case of a 35-year-old female who presented to the otolaryngology (ENT) department of the Central Military Emergency University Hospital in Bucharest, Romania, in February 2025. She complained of recurrent episodes of dizziness triggered by motion lasting for 4 years and worsening in the past 9 to 12 months. She described brief spells of vertigo prior to current complaints but was not able to provide any additional details about these spells and did not seek any medical advice at the time. These episodes were described as brief, lasting no longer than four to five minutes, and were consistently accompanied by nausea. During more recent dizzy episodes, continued movement progressively exacerbated her instability, culminating in a near-falling sensation; therefore, she was compelled to stop and wait for the imbalance to subside. The patient reported that symptoms were provoked by abrupt movements of the head or body, walking on unstable surfaces such as floating floors or suspension bridges, transitions between differing textures of flooring (carpet to hardwood), and physical activity on devices such as elliptical trainers or steppers. Symptom exacerbation was also noted during vertical oscillatory movements, such as when accompanying her child on a trampoline. She had no known allergies and was not under any chronic medication for the past years. Her only previous medical record pointed out that in 2021 she had undergone a right partial parotidectomy with a functional jugulocarotid neck dissection for the resection of a parotid tumor, later diagnosed histologically as a pleomorphic adenoma with epithelial, cartilaginous, and myxoid components. Current ENT examination was unremarkable except for the presence of a right-sided cervical surgical scar with mild asymmetry noted between the right and left lateral cervical regions, consistent with post-surgical anatomical changes (Figure 1). The patient complained that due to aggravated imbalance significantly her ability to engage in daily activities, including accompanying her child to playgrounds and exercising at the gym, was severely limited. She also described experiencing episodes of anxiety in visually complex environments, such as supermarkets, episodes induced by the fear that she would become dizzy and nauseous.

A complete audio-vestibular evaluation was performed during the initial clinical examination. PTA and tympanometry were conducted using the Callisto^TM^ and Titan^TM^ platforms (Interacoustics A/S, Middelfart, Denmark). Vestibular screening and ocular motor tests were performed using VisualEyes^TM^ (VN415 and VF405) software and infrared cameras. (Interacoustics A/S, Middelfart, Denmark).

cVEMP and oVEMP were recorded with the Eclipse^TM^ system (Interacoustics A/S, Middelfart, Denmark), using insert earphones and provided protocols (Table 1). The optimal electrode placement and recording techniques for recording cVEMPs using the trapezius muscle involve specific considerations regarding electrode positioning. The trapezius muscle, particularly the upper and middle fibers, can be used for recording cVEMPs [27], although traditionally, the sternocleidomastoid (SCM) muscle is more commonly used. In the case of cVEMP testing, recordings were made from the trapezius muscle on the affected side due to partial absence and surgical denervation of the SCM muscle. We used the midpoint between the C7 spinous process and the lateral margin of the acromion for electrode placement. A simple yet effective test, the ‘bucket test’, was also performed on the patient, in order to assess the subjective vertical visual [28,29].

Video Head Impulse Testing (vHIT) was performed using the EyeSeeCam^TM^ (version 1.2.0) vHIT system (Interacoustics A/S, Middelfart, Denmark), with high-speed infrared eye tracking focused on the left eye. All six semicircular canals were assessed individually by delivering brief, passive, high-acceleration head impulses in the horizontal and vertical planes. Gain values and presence or absence of corrective saccades were recorded to evaluate the functional integrity of the vestibulo-ocular reflex (VOR).

Posturographic assessment was performed using the PhysioSensing^®^ platform (Sensing Future Technologies, Coimbra, Portugal), a static posturography device designed to quantify balance performance under systematically altered sensory conditions. The evaluation protocol consisted of four standardized conditions assessing the subject’s ability to maintain postural stability while progressively challenging visual and somatosensory inputs:

1. Firm surface, eyes open—All sensory inputs available; baseline condition;
2. Firm surface, eyes closed—Removes visual input; assesses somatosensory and vestibular reliance;
3. Foam surface, eyes open—Somatosensory input is altered; evaluates ability to rely on visual and vestibular cues;
4. Foam surface, eyes closed—Removes visual input and alters somatosensory cues; primarily assesses vestibular contribution.

For each condition, the mean center of pressure (COP) sway velocity (°/s) was calculated, with lower values indicating better postural stability. A composite sway velocity score was also derived, representing the subject’s overall balance performance across all conditions.

The patient underwent brain MRI using a 1.5 Tesla Magnetom Sempra scanner (Siemens Healthineers, Erlangen, Forchheim, Germany). The protocol included high-resolution sequences targeting the posterior fossa and cerebellopontine angle, comprising T1-weighted, T2-weighted, fluid-attenuated inversion recovery (FLAIR), and other dedicated sequences relevant for posterior fossa evaluation, both before and after contrast administration, to assess for potential retrocochlear pathology.

The DHI was administered to evaluate the impact of symptoms on the patient’s daily life. The DHI is a validated, self-assessment questionnaire [30], comprising 25 items grouped into three subscales: functional, emotional, and physical. Each item is scored as *Yes* (4 points), *Sometimes* (2 points), or *No* (0 points), with a total possible score ranging from 0 to 100, where higher scores indicate a greater perceived handicap. The questionnaire was completed at baseline and at the 3-month follow-up, after completing the treatment and vestibular rehabilitation program.

**Figure 2:**
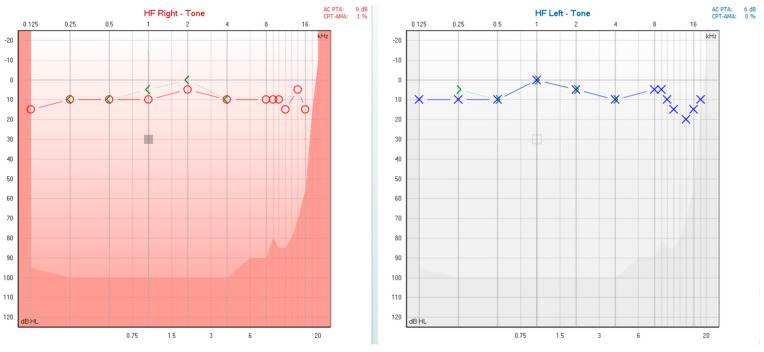
PTA showing bilateral normal hearing thresholds with extended frequency range up to 16 kHz.

During the initial patient evaluation, audiological assessment (PTA) revealed normal hearing thresholds bilaterally (Figure 2). Tympanometry and acoustic stapedial reflex testing yielded normal results (bilateral type A tympanograms and present acoustic stapedial reflex), indicating a normal functioning middle ear [31]. The patient did not experience any dizziness or vertigo during tympanometry and acoustic stapedial reflex testing, removing clinical suspicions of a third mobile window pathology [32].

The DHI score upon initial assessment was 24, indicating mild perceived disability. The emotional subscale was the most affected (12 points), reflecting frustration and anxiety related to symptom unpredictability, followed by the functional subscale (6 points), with minimal impact on the physical subscale (6 points).

**Figure 3:**
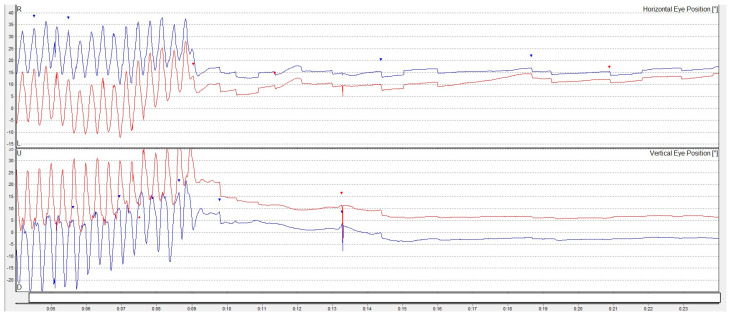
Transient, low-amplitude, left-beating horizontal nystagmus, with no vertical component, identified after the horizontal head-shaking test.

Objective vestibular assessment was conducted using VNG. No spontaneous or gaze-evoked nystagmus was observed, and oculomotor function remained intact. No typical positional nystagmus was detected, excluding suspicions of benign positional paroxysmal vertigo. Vibration-induced nystagmus was absent. However, a weak, transient, left-beating horizontal nystagmus was obtained after the horizontal head-shaking test (Figure 3) as well as in certain head extension positions during positional testing, which was interpreted as a sign of vestibular function asymmetry, possibly reflecting a poorly compensated unilateral vestibular deficit [33]. The subjective visual vertical test (‘bucket test’) showed no deviation (0°).

**Figure 4:**
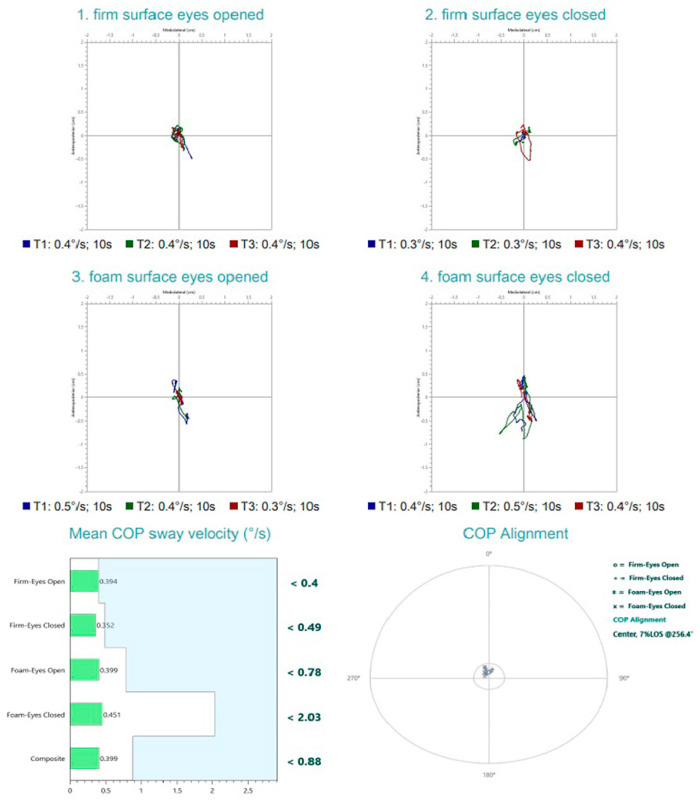
Posturography test results upon initial evaluation. Normal postural control with no evidence of vestibular, proprioceptive, or visual integration deficits.

**Figure 5:**
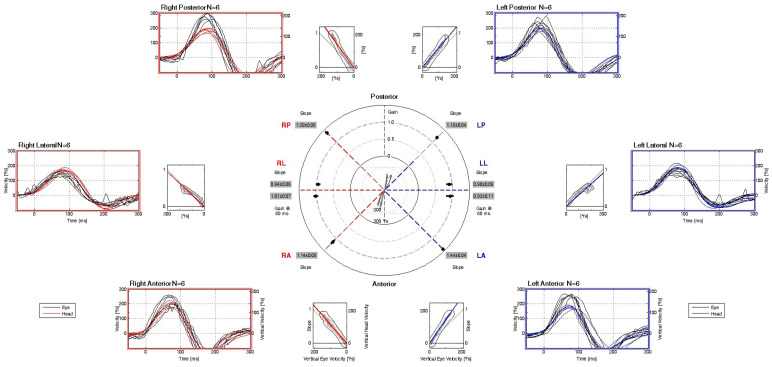
vHIT result. Normal gain values and no corrective saccades.

Posturographic assessment demonstrated preserved balance performance across all four testing conditions, with mean COP sway velocities falling within normative reference limits. These findings indicate intact somatosensory, visual, and vestibular contributions to postural stability under both firm and foam surface conditions, with eyes open and closed. However, the COP traces revealed a noticeable degree of sway, particularly under altered sensory conditions, suggesting subtle instability despite overall normal composite sway velocity scores. This pattern may reflect a subclinical vestibular or sensory integration vulnerability not captured by gross performance metrics alone. (Figure 4). The vHIT recordings demonstrate normal vestibulo-ocular reflex (VOR) function across all six semicircular canals. Eye velocity traces closely match head velocity traces, with VOR gains within physiological limits (0.90–1.44) for each canal. No overt or covert corrective saccades are evident in any canal plane, indicating preserved high-frequency vestibular function bilaterally. (Figure 5).

**Figure 6:**
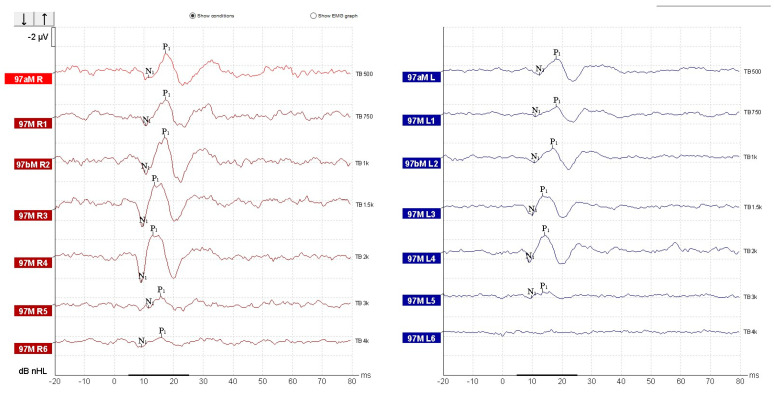
Recorded oVEMP responses.

oVEMPs testing demonstrated reproducible N1–P1 waveforms bilaterally, with latencies within normal limits (N1 ~ 10 ms, P1 ~ 15 ms), showing maximal amplitudes at 500–750 Hz and progressive reduction at higher frequencies (Figure 6). Mild interaural amplitude difference was observed; however, it did not exceed 30% and therefore was interpreted as within normal limits. These findings are consistent with preserved bilateral utricular function.

**Figure 7:**
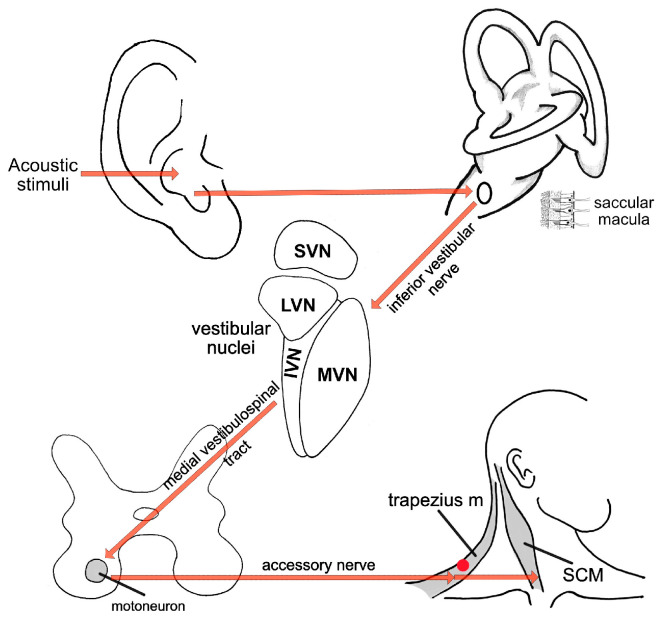
Schematic representation of the neural pathway underlying cVEMP testing and recording. The vestibular nuclei are represented by the superior (SVN), the lateral (LVN), medial (MVN), and inferior (IVN) subdivisions. The red dot on the trapezius muscle border indicates electrode placement.

In contrast, cVEMPs showed an abnormal result. In the left ear, reproducible biphasic P1–N1 waveforms were observed across all frequencies, with normal latencies (P1 ~ 13 ms, N1 ~ 23 ms) and amplitudes within the expected normative range. On the right side, responses were present but demonstrated a marked amplitude reduction compared to the contralateral side, most notable at 500 Hz (Figure 7). The interaural amplitude difference was greater than 50%, indicating right-sided saccular hypofunction. Due to the prior cervical surgery and partial denervation and scarring of the right SCM muscle, standard electrode placement and muscle activation were not ideal. Given the suspicion of right-sided vestibular dysfunction raised by the horizontal head-shaking test and positional testing nystagmus, alternative cVEMP recording was done using the superior descending fibers of the trapezius muscle [27]. Although normative data for cVEMP derived from trapezius muscle contraction are not well represented in the literature, we chose to record cVEMP from the trapezius muscle guided by the following reasoning:

1. Post-surgical cervical scarring and asymmetry with denervation and surgically absent SCM muscle fibers;
2. Common innervation for the trapezius muscle and SCM muscle (Figure 7) [34];
3. Some existing literature data for results comparison [27,35,36].

**Figure 8:**
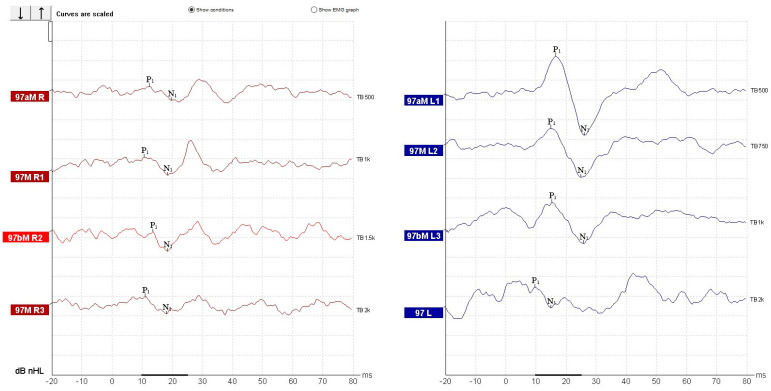
Trapezius recorded cVEMP response.

**Figure 9:**
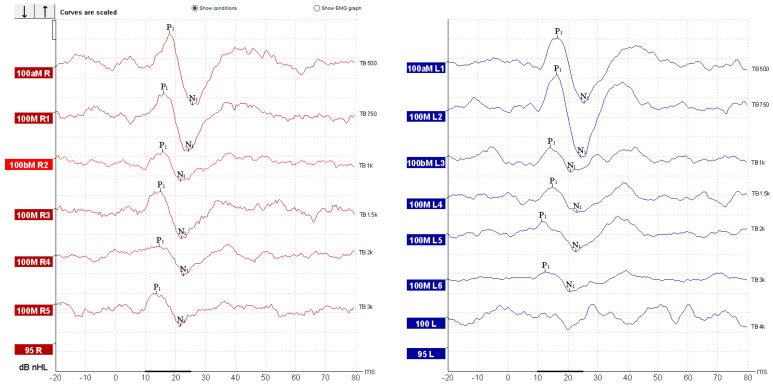
Normal cVEMP response recorded from the SCM. Healthy control subject.

For cVEMP recording, we had the patient sit with the head rotated contralaterally to the recording site and with the ipsilateral shoulder slightly raised. The presence and symmetry of responses were used as surrogate indicators. We used the same latency intervals for wave identification as we would in SCM recorded cVEMP [37]. Recordings revealed robust cVEMP responses on the left side, but significantly reduced amplitudes on the right, yielding an asymmetry ratio of 0.69 (69%) (Figure 8). The trapezius-recorded cVEMP shows reproducible biphasic N1P1 complexes with latency within the normal range, consistent with the expected temporal profile of standard SCM-based cVEMP responses (Figure 9). The wave morphology follows the classic inhibitory pattern. These findings were interpreted as evidence of right-sided sacculo-collic hypofunction [38], in the absence of semicircular canal impairment and in accordance with existing literature data and technical specifications provided by the manufacturer of the testing equipment [12,39]. We reported and evaluated the recorded waveforms to other normal amplitude and latency waveforms recorded using the same system and settings in healthy and normal vestibular subjects (Figure 9).

All current findings indicated a right chronic vestibular deficit, without proper vestibular compensation. The patient was diagnosed with an isolated right-sided saccular dysfunction (hypofunction). Oral treatment with betahistine was initiated (48 mg per day) and our patient was enrolled in a tailored vestibular rehabilitation program. The program incorporated graded vertical stimulation exercises designed to improve saccular sensitivity and function [40]. The program was based on:

- Low-frequency vertical oscillations on a fitness ball;
- Higher-frequency bouncing on a trampoline;
- Speed-varied vertical movements on a rocker board.

After four weeks, the patient reported subjective improvement. Nausea no longer accompanied the therapeutic movements, and dizziness was no longer provoked by previously triggering activities. She did, however, report transient sensations of unsteadiness and “rubbery legs” immediately following the exercises. Motivated by progress, the patient independently engaged in more intensive vestibular stimuli, culminating in a prolonged instability episode lasting approximately two hours after a carousel ride. Despite this, the patient continued the treatment and vestibular rehabilitation for the whole 3 months.

**Figure 10:**
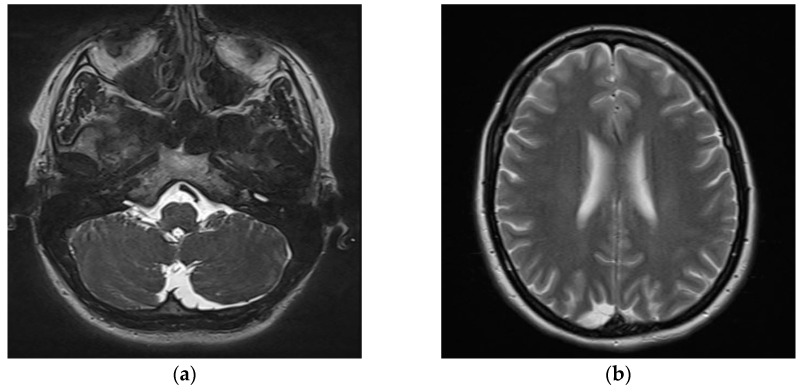
MRI scan showing: (**a**) Axial T2-weighted MRI of the posterior fossa and cerebellopontine angle; (**b**) Axial T2-weighted MRI of the cerebral hemispheres.

Subsequent contrast-enhanced MRI of the cervical spine and brain was performed to monitor post-surgical outcomes and to exclude central etiologies in between the two evaluations. Imaging identified two incidental arachnoid cysts: a left retrovermian parasagittal cyst measuring 18 × 21 mm, and a smaller right paramedian-parietal cyst measuring 11 × 21 mm (Figure 10). These findings were deemed clinically insignificant in relation to the patient’s vestibular symptoms on a further neurological examination.

**Figure 11:**
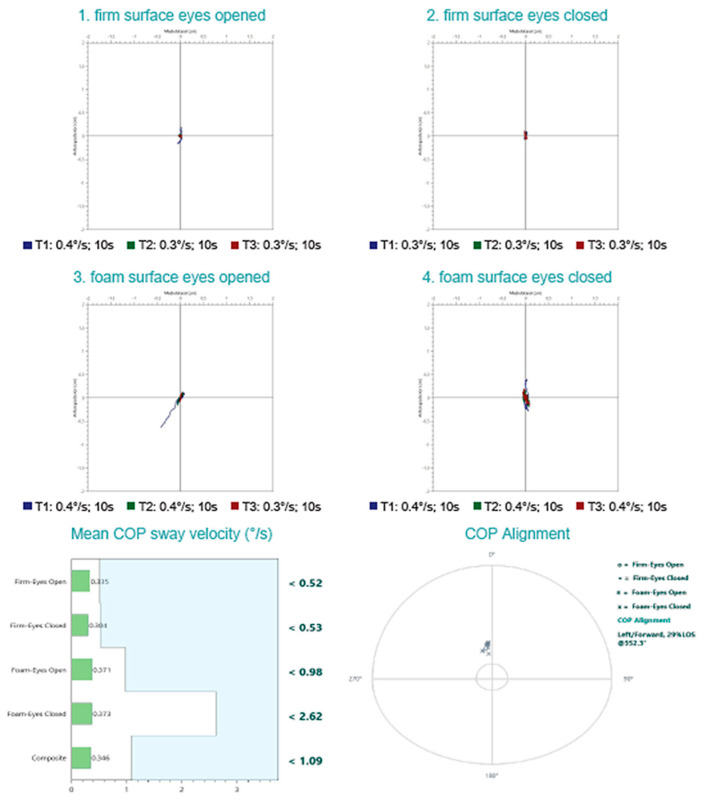
Posturography test results at 3 months. Normal postural control with improvement in postural control (less swaying) across tested conditions.

At the 3-month follow-up evaluation, the patient reported no recurrent dizziness or instability. Audiometric testing and tympanometry revealed normal hearing thresholds and middle ear function. No spontaneous, positional or post-head-shaking nystagmus was detected during the infrared video examination. At the 3-month reassessment, overall postural stability improved, with a reduction in mean COP sway velocity from 0.366°/s to lower composite values, and shorter, more centralized COP traces in all test conditions. Notably, the foam surface eyes-closed condition—primarily reliant on vestibular input—showed the greatest improvement, with reduced directional drift and tighter sway patterns (Figure 11). cVEMP testing, however, continued to demonstrate asymmetry. The patient was reevaluated with the DHI, scoring 6 points, indicating minimal to absent perceived disability. Satisfied with her recovery and symptom resolution, she was advised to discontinue further treatment and be rescheduled for evaluation in one year.

## 3. Discussion

Although this report provides data into the use of trapezius recorded cVEMP in subjects where the SCM is anatomically compromised, several limitations must be acknowledged. This remains a single case report, with no comparable cases identified in the literature to date. Current experience with standardized cVEMP recording from the trapezius muscle is limited. Further research on a larger cohort would be necessary to validate this adaptation as standard practice and establish additional normative reference data for the inclusion of trapezius recorded cVEMP in clinical protocols.

In the dedicated literature, reports on the etiology of vestibular disorders and the corresponding number of affected patients, are a welcome contribution for all practitioners. However, in all reports, a number of patients complaining of dizziness, vertigo or postural imbalance remain undiagnosed [41,42]. Accurate diagnosis and differentiation between central and peripheral vestibular disorders are essential for establishing effective management strategies and optimizing clinical patient outcomes. Central vestibular pathology may originate within the brainstem or cerebellum, whereas peripheral lesions involve the vestibular labyrinth or vestibular nerve [43]. Misclassification may result in suboptimal therapeutic interventions, delayed recovery, or unnecessary investigations, thereby adversely affecting patient prognosis [44].

Vertigo and dizziness lead to increased healthcare services across various sectors, including inpatient, outpatient, and emergency services. In the United States, the mean incremental annual healthcare expenditure directly associated with these conditions is approximately 2658.73 dollars per patient, with total annual expenditures reaching 48.1 billion dollars [45]. Diagnostic imaging, particularly neuroimaging such as computed tomography (CT) and MRI scans, is a major cost driver. In 2011, neuroimaging accounted for about 12% of the total costs for dizziness visits, with CT scans costing 360 million and MRI 110 million [46]. In Germany, individuals with vertigo incur an additional EUR 818 in healthcare costs annually compared to those who do not experience vertigo or dizziness [47]. Vertigo significantly impacts work productivity, with a large proportion of affected individuals reducing their workload, losing workdays, or even changing or quitting jobs due to symptoms. In a multi-country study, 69.8% of employed patients with vertigo reported reduced workload, and 63.3% had lost working days [48]. The societal burden of vertigo is even greater in the aging population, leading to increased demand for healthcare services and assistance in daily activities. This burden is reflected in decreased productivity and increased need for social support [49].

Recent literature reviews cite peripheral vestibular disorders as consistently more frequently diagnosed and reported than central vestibular lesions, although central disorders are the more critical exclusion diagnosis due to their potential severity [50,51]. This reinforces the clinical importance of accurate differential diagnosis between the two entities using vestibular function tests and imaging when needed. Vestibular lesions or partial disfunction is prevalent among patients experiencing dizziness, with studies indicating that a significant portion of these patients exhibit some form of vestibular abnormality. For instance, a study involving 1116 patients found that a majority had at least one abnormal vestibular test result, with older adults showing higher rates of dysfunction in saccadic and horizontal tracking eye movements, as well as positional testing [52]. In a South Korean population, the prevalence of vestibular dysfunction was noted to be 1.84%, with dizziness being more common in older adults and associated with factors such as hearing loss and stress [53].

The timing of vestibular function testing is crucial for the accurate diagnosis and effective treatment of vestibular disorders. Early vestibular function testing can help distinguish between peripheral and central vestibular disorders, which is critical for determining the appropriate treatment pathway. For instance, vHIT and cVEMP are effective for an accurate diagnosis between central and peripheral disorders [54]. Despite this, general access to such vestibular tests is not widespread. The accessibility and availability in primary care settings as well as emergency care are influenced by several factors, including cost, technical requirements, and the level of expertise needed to interpret the results [55,56], taking these tests outside these settings and into specialized clinics or centers [57,58]. Even in specialized clinical settings, time remains a critical and often constraining factor. The complexity of vestibular disorders significantly influences the duration and scope of vestibular exams and associated tests. The duration of vestibular testing can be substantial, often taking more than one hour [59]. Existing literature points out that a routine balance function examination, including rotational chair examination, electronystagmography, cVEMP and oVEMP, typically lasts about 2.5 h [60]. Even when certain vestibular assessments are not needed or replaced with others which might be easier to perform, the overall duration of clinical examination and vestibular evaluation remains substantial, thereby limiting the number of patients that can be assessed per clinician.

Going through available literature, we may conclude that an accurate diagnosis for the proper treatment of vestibular disorders is essential, particularly in high-volume clinical settings [61] and especially in patients with unclear symptoms [62]. Early differentiating central from peripheral causes is critical [63] to selecting effective therapies, reducing unnecessary interventions, and lowering healthcare costs [64]. Comprehensive vestibular testing (cVEMP, oVEMP, vHIT, CDT, audiometry, and validated questionnaires such as the DHI) enhances diagnostic accuracy, supports timely intervention [65], and promotes better patient recovery outcomes [66].

Saccular dysfunction, often associated with vestibular disorders such as MD, presents specific clinical features and diagnostic criteria [67]. The primary diagnostic tool for assessing saccular function is the cVEMP test, which evaluates the sacculus’s response to sound stimuli. This test is particularly useful in detecting saccular dysfunction in patients with MD, where it correlates with low-frequency hearing loss but not with canal paresis [67]. Individuals may exhibit poor postural performance, especially in dynamic conditions, indicating a reliance on visual cues for balance [67]. Dizziness and vertigo are common symptoms associated with vestibular dysfunction, including saccular impairment, and can significantly impact daily activities [68]. cVEMP is the primary diagnostic test for saccular dysfunction. An absent or asymmetric response on the affected side is indicative of saccular impairment [67]. Pure tone audiometry is used to assess hearing loss, which, particularly at low frequencies, correlates with saccular dysfunction [67]. Posturography tests evaluate balance and postural control, helping to identify patients with saccular dysfunction who may be visually dependent for balance [67]. Saccular dysfunction can coexist with other vestibular pathologies, such as BPPV, especially following concussions [68].

While the SCM remains the standard recording site for cVEMPs, accumulating evidence supports the use of alternative recording sites when SCM activation is compromised. Studies have demonstrated that other cervical or shoulder muscles—such as upper trapezius or posterior cervical muscles—can yield identifiable cVEMP waveforms, provided that muscle contraction is adequately monitored and standardized [69,70,71]. In particular, when anatomical or surgical factors prevent reliable SCM contraction, recording from an alternative muscle can still provide valid information about saccular-inferior vestibular nerve function, provided normative data or side-to-side comparisons are available [72]. Thus, in selected cases, non-SCM cVEMP recordings may serve as an appropriate surrogate, allowing vestibular lesion localization when conventional SCM recordings are not feasible.

Diagnosing and monitoring vestibular disorders requires a comprehensive approach that can integrate more than one vestibular test. The most effective tests include vHIT [69], cVEMP and oVEMP [73], and VNG [74]. These tests are non-invasive and provide a detailed assessment of the vestibular system, covering all five vestibular analyzers. Each test has unique strengths and applications, making them essential tools in both clinical and research settings. One of the primary methods for diagnosing and monitoring saccular disfunction is cVEMP testing [12,67]. Although canal dysfunction remains the typicial trigger for spinning vertigo, isolated otolith organ impairment—limited to the saccule and/or utricle—can present imbalance, motion-provoked unsteadiness, tilt/translation sensations, or visually complex-induced disequilibrium in patient where routine canal tests are normal. Recent data on isolated otolith dysfunction emphasizes that the diagnosis hinges on abnormal cVEMP and/or oVEMP indicating saccular or utricular involvement, with canal function tests (vHIT) remaining normal [12]. Practice guidelines concur on test attribution: cVEMP reflects saccular–inferior vestibular nerve pathways and oVEMP reflects utricular–superior vestibular nerve pathways. Their complementary use with vHIT may be able to topographically separate disorders [68]. Accordingly, in rare but clinically meaningful cases, isolated saccular or utricular dysfunction can underlie imbalance, and a VEMP-plus-vHIT test battery provides the required specificity to confirm otolith-limited disease while ruling out semicircular canal deficits [12,75].

In addition to vestibular objective testing, questionnaires can also be used to monitor patients’ recovery and symptom impact. The DHI is a validated instrument for measuring vestibular symptoms and their impact on patients’ quality of life. It has been shown to be effective in evaluating symptoms before and after treatments such as stereotactic radiosurgery for vestibular schwannomas, with significant improvements in DHI scores observed post-treatment [25]. The DHI is also used for quality control in the treatment of peripheral vestibular dysfunctions, demonstrating significant reductions in dizziness complaints post-therapy, thus confirming its utility in therapy evaluation and monitoring [76]. CDT may also be used in conjunction with previous cited methods to monitor vestibular disfunctions [77]. Posturography, particularly CDP, offers several advantages in diagnosing vestibular dysfunction compared to other diagnostic methods. CDP evaluates the integration of visual, vestibular, and somatosensory inputs, providing a more integrated view of balance control, unlike other tests that focus on specific components of the vestibular system [78]. Unlike other subjective tests, CDP provides objective data on balance performance, which can be quantified and documented graphically and numerically [79], and thus monitored between patient evaluations.

cVEMP are typically measured in the SCM to assess saccular and inferior vestibular nerve function. The trapezius muscle, due to its accessibility, size and common nerve supply, presents a potential site for recording these evoked potentials, although it is less commonly used compared to the SCM. Research comparing cVEMP responses from the SCM and trapezius muscles indicates that while both muscles can exhibit vestibular-evoked potentials, the responses may differ in amplitude and symmetry [80]. For patients where the SCM cannot provide optimal electrical signal input, the trapezius muscle may be used, considering that the obtained action potentials should be analyzable within certain predetermined criteria in order to be useful for diagnosis [26,81].

MRI is a valuable tool in the diagnosis of vestibular disorders, but it is not typically used as a standalone diagnostic method. MRI is an integral part of a multimodal comprehensive diagnostic approach for vestibular disorders [82]. It is used alongside clinical evaluations and other tests to provide a comprehensive assessment of the patient’s condition, helping with differential diagnosis but rarely providing a complete diagnosis when used solitary [83]. While MRI is often used to detect structural abnormalities, its effectiveness can differ based on the location of the vestibular dysfunction. The 3D-FLAIR MRI imaging technique has shown promise in detecting unilateral peripheral vestibular dysfunction. In a study, nearly half of the patients with this type of lesion exhibited abnormal MRI findings, such as endolymphatic hydrops and perilymphatic enhancement (which are common in MD), which were correlated with cochleovestibular function test results. The study highlighted that MRI findings were positively correlated with hearing impairment and vestibular dysfunction, suggesting a high sensitivity and specificity for peripheral abnormalities when specific imaging protocols are used [84]. The same imaging technique has improved the sensitivity and specificity of diagnosing perilymphatic fistulas, a condition that can mimic other vestibular disorders. The presence of a ‘round window sign’ on MRI has been identified as a sensitive and specific indicator of this type of fistula, aiding in pre-operative diagnosis [85]. For central etiology MRI is less sensitive in detecting central vestibular lesions, especially in acute settings. Clinical examinations may outperform MRI in identifying central lesions, such as brainstem strokes, within the first 48 h of symptom onset. This is due to the subtlety of central lesions and the limitations of MRI in capturing acute ischemic changes in the brainstem and cerebellum [6]. Most often, MRI scans turn up ‘incidentalomas’, benign lesions which are not directly related and cannot accurately explain patient symptoms [86].

Vestibular rehabilitation (VR) is a therapeutic approach designed to address balance disorders, particularly those related to peripheral vestibular syndromes [87]. The effectiveness of VR in treating saccular-related balance disorders is supported by various studies, which highlight its role in improving balance, reducing symptoms of dizziness, and enhancing the quality of life for patients with vestibular disorders. VR is particularly effective due to its ability to promote vestibular compensation, a process that helps the brain adapt to changes in the vestibular system [88]. VR employs exercises that target the vestibular, visual, and proprioceptive systems to improve balance and reduce dizziness [88]. Techniques such as substitution, adaptation, habituation, and compensation are used to facilitate vestibular compensation, which is essential for managing saccular-related balance disorders. The exercises are tailored to individual needs, ensuring that the rehabilitation program is effective for each patient [89,90]. Studies have shown that VR significantly improves balance and reduces the risk of falls in patients with vestibular disorders, including those with saccular dysfunction [91,92].

Betahistine is widely used in the treatment of peripheral vestibular lesions, such as those seen in MD and BPPV. The efficacy and safety of betahistine have been evaluated in various clinical settings, demonstrating its potential to improve symptoms and quality of life in affected patients. Betahistine has been shown to significantly improve symptoms associated with peripheral vestibular vertigo [93]. In a real-life study, patients experienced an average improvement of 56.6 points in vertigo symptoms after the first week, reaching 89.3 points by week 12, with 73% achieving complete improvement by the end of the study [94]. Another study demonstrated a significant reduction in the DHI scores by 32 points over 12 weeks, indicating substantial symptom relief [95] and a good first treatment choice with few side-effects as pointed out by other studies [96].

## 4. Conclusions

Accurate vestibular diagnosis is essential for prescribing effective treatment and avoiding the misdiagnosis and misclassification of patients under idiopathic etiologies, particularly in rare, isolated lesions such as isolated saccular dysfunctions. This case presents the importance of a comprehensive, extended vestibular evaluation for accurate lesion localization. The combination of PTA and tympanometry confirmed preserved cochlear and middle ear integrity, while vHIT and oVEMP demonstrated normal canal and utricular function. In contrast, the trapezius-recorded cVEMP revealed clear amplitude asymmetry consistent with right-sided saccular hypofunction. MRI was essential in excluding central vestibular pathology and possible structural postoperative complications. Furthermore, DHI reflected the burden of imbalance while helping to exclude persistent postural-perceptual dizziness (PPPD) as a primary diagnosis through its pattern of improvement following targeted vestibular rehabilitation [97]. Together, these complementary tools provided a complete topographic and functional profile of the vestibular system, enabling precise localization of the lesion and guiding personalized treatment options.

This individualized approach, supplemented by validated symptom questionnaires such as the DHI and functional balance assessment through posturography, facilitated optimal treatment monitoring. In line with presented findings, our results emphasize that a custom-tailored, multi-approach treatment strategy not only guides appropriate rehabilitation but also contributes to improved quality of life, reduced healthcare costs, and optimal outcomes for patients with complex or atypical vestibular presentations.

Although this report describes a single, atypical case, it provides valuable information which may contribute to further understanding of other rare clinical presentations in which conventional vestibular testing is not feasible. Despite its limitations, this case highlights that even isolated and uncommon patient scenarios may broaden current understanding and encourage further research on the feasibility and diagnostic reliability of trapezius-recorded cVEMP in selected clinical contexts.

## Figures and Tables

**Table 1:** Stimulus and recording parameters for cVEMP and oVEMP testing.

Parameter	cVEMP	oVEMP
Stimulus type	Tone Burst	Tone Burst
Stimuli per second	5.1/s	5.1/s
Polarity	Rarefaction	Rarefaction
Test frequency	500 Hz (optional: 250, 750, 1000 Hz)	500 Hz (optional: 250, 750, 1000 Hz)
Rise/Fall time	2 cycles (4.0 ms)	2 cycles (4.0 ms)
Plateau	2 cycles (4.0 ms)	2 cycles (4.0 ms)
Stimulus transducer	Insert earphones (ER-3A or equivalent)	Insert earphones (ER-3A or equivalent)
Stimulus ear	Ipsilateral	Ipsilateral
Masking	Off	Off
Recording site	Trapezius muscle (ipsilateral)	Inferior oblique muscle (contralateral)
Patient position	Supine with head inclined ipsilaterally	Reclined with upward gaze
Intensity	97 dB nHL and descend	97 dB nHL and descend
Recording window	−20 ms to 80 ms	−20 ms to 80 ms
Number of stimuli	200	500
Filter settings	Low pass: 1000 Hz; High pass: 10 Hz (6 dB/oct)	Low pass: 1000 Hz; High pass: 10 Hz (6 dB/oct)
Rejection level	±800 µV (66 dB)	±400 µV (72 dB)
Display scale	20 µV/div	10 µV/div
Rejection level	±800 µV (66 dB)	±400 µV (72 dB)
Display scale	20 µV/div	10 µV/div

## Data Availability

The original contributions presented in this study are included in the article. Further inquiries can be directed to the corresponding author.

## Abbreviations

The following abbreviations are used in this manuscript:

DHI	Dizziness Handicap Inventory
PTA	Pure Tone Audiometry
VNG	Videonistagmography
cVEMP	Cervical Vestibular Evoked Myogenic Potentials
oVEMP	Ocular Vestibular Evoked Myogenic Potentials
CDP	Computed Dynamic Posturography
MRI	Magnetic Resolution Imaging
MD	Ménière’s Disease
BPPV	Benign Paroxysmal Positional Vertigo
SSCD	Superior Semicircular Canal Dehiscence
SSNHL	Sudden Sensorineural Hearing Loss
COP	Centre of Pressure
FLAIR	Fluid Attenuated Inversion Recovery
ENT	Otorhinolaryngology
SCM	Sternocleidomastoid
SVN	Superior Vestibular Nucleus
LVN	Lateral Vestibular Nucleus
MVN	Medial Vestibular Nucleus
IVN	Inferior Vestibular Nucleus
CT	Computed Tomography
VR	Vestibular Rehabilitation

## References

[1] Strupp M., Brandt T. (2025). Acute Unilateral Vestibulopathy (AUVP). Oxford Textbook of Vertigo and Imbalance.

[2] Dieterich M., Strupp M., Brandt T. (2025). Bilateral Vestibulopathy. Oxford Textbook of Vertigo and Imbalance.

[3] Kudo Y., Johkura K. (2023). Vertigo and Dizziness Due to Cerebrovascular Disease. Equilib. Res..

[4] Dos Santos Carvalho M., Oliveira A.C., de Oliveira Almeida B.M., Chon C.W., de Alcantara Machado D.A., Neto E.P.M., Brazilian F.S., Chaves F.N.R., Centenaro G.D., Schug G.H. (2024). Central and Peripheral Vertigo: Neurological and Otorhinolaryngological Approaches. Challenges and Research in Health Sciences: A Multidisciplinary Approach.

[5] Kitazawa M., Morita Y., Yagi C., Takahashi K., Ohshima S., Yamagishi T., Izumi S., Koizuka I., Horii A. (2021). Test Batteries and the Diagnostic Algorithm for Chronic Vestibular Syndromes. Front. Neurol..

[6] Thömke F. (2018). Acute Vestibular Syndrome: Clinical Examination Outperforms MRI in the Detection of Central Lesions. Nervenarzt.

[7] Mattingly J.K., Riggs W.J., Adunka O.F. (2019). Vestibular Evoked Myogenic Potentials. Diagnosis and Treatment of Vestibular Disorders.

[8] Bansal S., Sahni S., Sinha S.K. (2014). Cervical and Ocular Vestibular Evoked Myogenic Potentials in Individuals with Severe to Profound Hearing Loss. J. Hear. Sci..

[9] Seo T. (2017). Clinical Application of VEMP. Equilib. Res..

[10] Young Y.-H. (2013). Potential Application of Ocular and Cervical Vestibular-Evoked Myogenic Potentials in Meniere’s Disease: A Review. Laryngoscope.

[11] Noij K., Rauch S. (2020). Vestibular Evoked Myogenic Potential (VEMP) Testing for Diagnosis of Superior Semicircular Canal Dehiscence. Front. Neurol..

[12] Murofushi T., Suh M.-W., Manzari L. (2022). Editorial: Isolated Otolith Dysfunction and Vertigo. Front. Neurol..

[13] Strupp M., Grimberg J., Teufel J., Laurell G., Kingma H., Grill E. (2020). Worldwide Survey on Laboratory Testing of Vestibular Function. Neurol. Clin. Pract..

[14] Magliulo G., Iannella G., Gagliardi S., Re M. (2015). A 1-Year Follow-up Study with C-VEMPs, O-VEMPs and Video Head Impulse Testing in Vestibular Neuritis. Eur. Arch. Oto-Rhino-Laryngol..

[15] Hannigan I.P., Rosengren S.M., Young A.S., Bradshaw A.P., Calic Z., Kwok B., Alraddy B., Gibson W.P.R., Kong J., Flanagan S. (2022). A Portrait of Menière’s Disease Using Contemporary Hearing and Balance Tests. Otol. Neurotol..

[16] Guo X., Xiao H., Lin C., Lin J., Cai H., Ke X., Lu Y., Ye S. (2024). Differentiating Definite and Probable Ménière Disease: A Comprehensive Evaluation of Audio-Vestibular Function Testing Combined with Inner Ear MRI. Otol. Neurotol..

[17] Jiang Z., Zhang J., Wang Y., Huang X., Yao Q., Feng Y., Huang S., Wang H., Yin S. (2021). Contribution of Audiogram Classification in Evaluating Vestibular Dysfunction in Sudden Sensorineural Hearing Loss with Vertigo. Front. Neurol..

[18] Jung S.G., Park J.W., Han S.Y., Park S.H., Nam S.I. (2013). The Role of Vestibular Function Tests in Patients with Sudden Sensorineural Hearing Loss Who Have Subclinical Vestibular Dysfunction. Korean J. Otorhinolaryngol.-Head Neck Surg..

[19] Castillo-Bustamante M., Anderson C., Gutierrez V. (2025). The Use of Posturography in Vestibular Evaluation of Neurodegenerative Disorders: Diagnostic and Rehabilitative Impacts. Cureus.

[20] Janc M., Sliwinska-Kowalska M., Politański P., Kaminski M., Józefowicz-Korczyńska M., Zamyslowska-Szmytke E. (2021). Posturography with Head Movements in the Assessment of Balance in Chronic Unilateral Vestibular Lesions. Sci. Rep..

[21] Strasilla C., Sychra V. (2017). Imaging-Based Diagnosis of Vestibular Schwannoma. HNO.

[22] Benezech L. (2023). The Hyperacute Vestibular Syndrome: Ear or Brain?. Lancet Neurol..

[23] Chen J., Guo Z., Wang J., Liu D., Tian E., Guo J., Kong W., Zhang S. (2022). Vestibular Migraine or Meniere’s Disease: A Diagnostic Dilemma. J. Neurol..

[24] Vanspauwen R., Knoop A., Camp S., van Dinther J., Offeciers F.E., Somers T., Zarowski A., Blaivie C. (2017). Outcome Evaluation of the Dizziness Handicap Inventory in an Outpatient Vestibular Clinic. J. Vestib. Res.-Equilib. Orientat..

[25] Rueß D., Vojacek S., Gungor E., Lüers J.-C., Hunsche S., Jabłońska K., Köcher M., Ruge M.I. (2025). Longitudinal Evaluation of Vestibular Symptoms in Patients with Vestibular Schwannoma After Robotic-Guided Stereotactic Radiosurgery Using the Dizziness Handicap Inventory (DHI). Stomatology.

[26] Chari D.A., Liu Y.-H., Chung J.J., Rauch S.D. (2021). Subjective Cognitive Symptoms and Dizziness Handicap Inventory (DHI) Performance in Patients with Vestibular Migraine and Menière’s Disease. Otol. Neurotol..

[27] Kim K.H., Shin S., Kim D.H. (2020). Optimal Trapezius Electrophysiological Recording Site. PM&R.

[28] Strupp M., Brandt T. (2009). Vestibular Neuritis. Semin. Neurol..

[29] Chang T., Winnick A., Hsu Y. (2019). The Bucket Test Differentiates Patients with MRI Confirmed Brainstem/Cerebellar Lesions from Patients Having Migraine and Dizziness Alone. BMC Neurol..

[30] Jacobson G.P., Newman C.W. (1990). The development of the Dizziness Handicap Inventory. Arch. Otolaryngol.-Head Neck Surg..

[31] Zwislocki J. (1982). Normal Function of the Middle Ear and Its Measurement. Audiology.

[32] Lazarou I., Sideris G., Papadimitriou N., Delides A., Korres G. (2025). Third Window Syndrome: An Up-to-Date Systematic Review of Causes, Diagnosis, and Treatment. J. Audiol. Otol..

[33] Striteska M., Valis M., Chrobok V., Profant O., Califano L., Syba J., Trnkova K., Kremlacek J., Chovanec M. (2022). Head-Shaking-Induced Nystagmus Reflects Dynamic Vestibular Compensation: A 2-Year Follow-up Study. Front. Neurol..

[34] Botts S.R., Yang S., Mansour M., Kaye A.M., Kaye A.D. (2024). Neck Anatomy. Anatomy, Back, Trapezius.

[35] Singh N., Kadisonga P., Ashitha P. (2014). Optimizing Stimulus Repetition Rate for Recording Ocular Vestibular Evoked Myogenic Potential Elicited by Air-Conduction Tone Bursts of 500 Hz. Audiol. Res..

[36] Rosengren S., Colebatch J. (2018). The Contributions of Vestibular Evoked Myogenic Potentials and Acoustic Vestibular Stimulation to Our Understanding of the Vestibular System. Front. Neurol..

[37] Satar B., Karababa E., Karaçaylı C. (2025). The Vestibular Evoked Myogenic Potentials (VEMPs) Test over the Trapezius Muscle: Neurophysiological Grounds in Muscle Extensor and Flexor Conditions. J. Int. Adv. Otol..

[38] Kingma C., Wit H. (2011). Asymmetric Vestibular Evoked Myogenic Potentials in Unilateral Menière Patients. Eur. Arch. Oto-Rhino-Laryngol..

[39] Interacoustics cVEMP Testing with Eclipse. https://www.interacoustics.com/abr-equipment/eclipse/support/cvemp-testing-with-eclipse.

[40] Hall C.D., Herdman S.J., Whitney S.L., Cass S.P., Clendaniel R.A., Fife T.D., Furman J.M., Getchius T.S.D., Goebel J.A., Shepard N.T. (2016). Vestibular Rehabilitation for Peripheral Vestibular Hypofunction: An Evidence-Based Clinical Practice Guideline. J. Neurol. Phys. Ther..

[41] Xing Y., Si L., Zhang W., Wang Y., Li K., Yang X. (2024). Etiologic Distribution of Dizziness/Vertigo in a Neurological Outpatient Clinic According to the Criteria of the International Classification of Vestibular Disorders: A Single-Center Study. J. Neurol..

[42] Xue H., Chong Y., Jiang Z.D., Liu Z.L., Ding L., Yang S.L., Wang L., Xiang W. (2018). Etiological Analysis on Patients with Vertigo or Dizziness. Natl. Med. J. China.

[43] Strupp M., Feil K., Zwergal A. (2021). Diagnosis and Differential Diagnosis of Peripheral and Central Vestibular Disorders. Laryngo-rhino-otologie.

[44] Grzesiak M., Carender W.J., Basura G.J. (2020). Posttraumatic Dizziness: Navigating the Maze Towards Accurate Vestibular Diagnosis and Treatment. Otol. Neurotol..

[45] Ruthberg J.S., Rasendran C., Kocharyan A., Mowry S.E., Mowry S.E., Otteson T.D., Otteson T.D. (2021). The Economic Burden of Vertigo and Dizziness in the United States. J. Vestib. Res.-Equilib. Orientat..

[46] Saber Tehrani A.S., Coughlan D., Hsieh Y.H., Mantokoudis G., Korley F.K., Kerber K.A., Frick K.D., Newman-Toker D.E. (2013). Rising Annual Costs of Dizziness Presentations to U.S. Emergency Departments. Acad. Emerg. Med..

[47] Wang X., Strobl R., Holle R., Seidl H., Peters A., Grill E. (2019). Vertigo and Dizziness Cause Considerable More Health Care Resource Use and Costs: Results from the KORA FF4 Study. J. Neurol..

[48] Benecke H., Agus S., Kuessner D., Goodall G., Strupp M. (2013). The Burden and Impact of Vertigo: Findings from the REVERT Patient Registry. Front. Neurol..

[49] Kovacs E., Wang X., Grill E. (2019). Economic Burden of Vertigo: A Systematic Review. Health Econ. Rev..

[50] Espinosa-Sanchez J.M., Lin C.C. (2025). Vestibular migraine. Front. Neurol..

[51] Perrin P., Gimmon Y. (2025). Reviews in neuro-otology: Highlighting recent advances in neuro-otology: New possibilities for future inquiries. Front. Neurol..

[52] Nimmo Z.M., Hwa T.P., Naples J.G., Shah R.R., Brant J.A., Eliades S.J., Bigelow D.C., Ruckenstein M.J. (2021). Age-Related Patterns of Vestibular Dysfunction in Dizziness and Imbalance: A Review of Vestibular Testing Results Among 1,116 Patients. Otol. Neurotol..

[53] Koo J.W., Chang M.Y., Woo S.-Y., Kim S., Cho Y.-S. (2015). Prevalence of Vestibular Dysfunction and Associated Factors in South Korea. BMJ Open.

[54] Chang T.P., Zee D.S., Kheradmand A. (2019). Technological Advances in Testing the Dizzy Patient: The Bedside Examination Is Still the Key to Successful Diagnosis. Dizziness and Vertigo Across the Lifespan.

[55] Kamal N., Taha H., Galal E. (2011). Office Vestibular Tests: A Battery Approach to Guide the Diagnosis of Dizzy Patients. Audiol. Med..

[56] Maarsingh O.R., van Vugt V.A. (2021). Ten Vestibular Tools for Primary Care. Front. Neurol..

[57] van de Berg R., Rosengren S.M., Kingma H. (2017). Laboratory Examinations for the Vestibular System. Curr. Opin. Neurol..

[58] Sandhu J.S., Sandhu J.S., Yung M., Parker-George J.C., Kearney B., Ray J. (2018). Assessment of Vestibular Function in Patients with Chronic Middle Ear Disease Using the VHIT and VEMP Test. Clin. Otolaryngol..

[59] Kelly E.A., Stocker C., Kempton C.M., Dierking D.M., Fehlberg H.E., Adams M.E. (2018). Vestibular Testing: Patient Perceptions, Morbidity, and Opportunity Costs. Otol. Neurotol..

[60] McCaslin D.L. (2008). Vestibular Testing: The Good, the Bad, and the Emesis Basin. Hear. J..

[61] Gizzi M., Rosenberg M.L. (1998). The Diagnostic Approach to the Dizzy Patient. Neurologist.

[62] Venhovens J., Meulstee J., Verhagen W.I.M. (2016). Acute Vestibular Syndrome: A Critical Review and Diagnostic Algorithm Concerning the Clinical Differentiation of Peripheral versus Central Aetiologies in the Emergency Department. J. Neurol..

[63] Liu X., Li Z., Ju Y., Zhao X. (2024). Application of Bedside HINTS, ABCD2 Score and Truncal Ataxia to Differentiate Cerebellar-Brainstem Stroke from Vestibular Neuritis in the Emergency Room. Stroke Vasc. Neurol..

[64] Phillips J.S., Newman J.L. (2023). Quantifying the Direct Cost Benefits of Vestibular Telemetry Using the CAVA System to Diagnose the Causes of Dizziness. Cost Eff. Resour. Alloc..

[65] Antonenko L.M., Parfenov V.A. (2016). A Specialized Approach to Diagnosing and Treating Vertigo. Neurol. Neuropsychiatry Psychosom..

[66] Futami S., Miwa T. (2024). Comprehensive Equilibrium Function Tests for an Accurate Diagnosis in Vertigo: A Retrospective Analysis. J. Clin. Med..

[67] de Waele C., Huy P., Diard J.P., Freyss G., Vidal P.-P. (1999). Saccular Dysfunction in Meniere’s Disease. Am. J. Otol..

[68] Mucha A., Fedor S., DeMarco D. (2018). Vestibular Dysfunction and Concussion. Handb. Clin. Neurol..

[69] Legois Q., Marx M., Paul A., Giraudet F. (2025). Vestibular evoked myogenic potentials beyond oVEMP and cVEMP: Exploring new recording sites. Clin. Neurophysiol..

[70] Lullo F., Piscosquito, Provitera V., Zamprotta L., Prisco C., Lanzillo B., Manganelli F., Santoro L., Nolano M. (2017). Myogenic Vestibular-Evoked Potentials: An Extension of the Assessment Protocol. Clin. Neurophysiol..

[71] Rosengren S.M., Weber K.P., Govender S., Welgampola M.S., Dennis D.L., Colebatch J.G. (2019). Sound-Evoked Vestibular Projections to the Splenius Capitis in Humans: Comparison with the Sternocleidomastoid Muscle. Appl. Physiol..

[72] Hougaard D.D., Abrahamsen E.R. (2019). Functional Testing of All Six Semicircular Canals with Video Head Impulse Test Systems. J. Vis. Exp..

[73] Albernaz P.L.M., Maia F.Z., Carmona S., Cal R., Zalazar G. (2019). Clinical Evaluation of the Vestibular System: The Vestibular Laboratory Tests. The New Neurotology.

[74] Tian E., Li F., Liu D., Wang J., Guo Z., Chen J., Guo J., Zhang S. (2023). Dispelling Mist That Obscures Positional Vertigo in Vestibular Migraine. Brain Sci..

[75] Park H.G., Lee J.-H., Oh S.H., Park M.K., Suh M.-W. (2019). Proposal on the Diagnostic Criteria of Definite Isolated Otolith Dysfunction. J. Audiol. Otol..

[76] Duong Dinh T.A., Wittenborn J., Westhofen M. (2021). The Dizziness Handicap Inventory for Quality Control in the Treatment of Vestibular Dysfunction. HNO.

[77] Gawronska A., Rosiak O., Pajor A., Janc M., Kotas R., Kaminski M., Zamyslowska-Szmytke E., Jozefowicz-Korczynska M. (2023). Instrumental and Non-Instrumental Measurements in Patients with Peripheral Vestibular Dysfunctions. Sensors.

[78] Furman J.M. (1994). Posturography: Uses and Limitations. Baillière’s Clin. Neurol..

[79] Di Fabio R.P. (1996). Meta-Analysis of the Sensitivity and Specificity of Platform Posturography. Arch. Otolaryngol.-Head Neck Surg..

[80] Ferber-Viart C., Duclaux R., Colleaux B., Dubreuil C. (1997). Myogenic Vestibular-Evoked Potentials in Normal Subjects: A Comparison between Responses Obtained from Sternomastoid and Trapezius Muscles. Acta Oto-Laryngol..

[81] Comacchio F., Zattoni G., Di Pasquale Fiasca V.M., Magnavita P., Bellemo B., Fasanaro E., Poletto E. (2025). Masseter Vestibular Evoked Myogenic Potentials (M-VEMPs) in Vestibular Neuritis. Audiol. Res..

[82] Jäger L., Strupp M., Brandt T., Reiser M.F. (1997). Imaging of the Labyrinth and Vestibular Nerve. Clinical Significance for Differential Diagnosis of Vestibular Diseases. Nervenarzt.

[83] Mafee M.F. (2000). Magnetic Resonance Imaging for Evaluation of Otic Labyrinth Pathology. Top. Magn. Reson. Imaging.

[84] Zhao T.T., Feng Y., Li X., Song N., Ma X., Sui R. (2023). The Value of Postcontrast Delayed 3D Fluid-Attenuated Inversion Recovery MRI in the Diagnosis of Unilateral Peripheral Vestibular Dysfunction. Quant. Imaging Med. Surg..

[85] Kemps G., Mistry J., Connor S., Obholzer R., Ainsworth C. (2023). Diagnosing Perilymphatic Fistula with 3D Flair MRI. Hear. Balance Commun..

[86] Wangaryattawanich P., Rutman A., Petcharunpaisan S., Mossa-Basha M. (2023). Incidental Findings on Brain Magnetic Resonance Imaging (MRI) in Adults: A Review of Imaging Spectrum, Clinical Significance, and Management. Br. J. Radiol..

[87] Sedeño-Vidal A., Hita-Contreras F., Montilla-Ibáñez M.A. (2022). The Effects of Vestibular Rehabilitation and Manual Therapy on Patients with Unilateral Vestibular Dysfunction: A Randomized and Controlled Clinical Study. Int. J. Environ. Res. Public Health.

[88] Chinese Geriatrics Society for Vestibular Disorders, Otolaryngology Expert Committee, National Health Commission Capacity Building and Continuing Education Center (2024). Expert Consensus on Vestibular Rehabilitation in Vestibular Disorders. Natl. Med. J. China.

[89] Alyahya D. (2022). A Systematic Review of Effectiveness of Vestibular Rehabilitation on Improving Balance in Patients with Different Conditions. Int. J. Physiother..

[90] Ferri N., Morone G., Piermaria J., Manzari L., Turolla A., De Tanti A., Ciancarelli I., Pillastrini P., Tramontano M. (2025). Knowledge, Barriers, and Future Directions of Vestibular Rehabilitation Practice in Neurorehabilitation: An Italian Survey. Healthcare.

[91] Tramontano M., Haijoub S., Lacour M., Manzari L. (2025). Updated Views on Vestibular Physical Therapy for Patients with Vestibular Disorders. Healthcare.

[92] ALShammari M., ALSharif D.S., Aldaihan M.M., Whitney S.L. (2025). Vestibular Rehabilitation in Saudi Arabia: Practice, Knowledge, and Beliefs of Physical Therapists. J. Clin. Med..

[93] Gameiro B., Fonseca A.C., Guimarães B.S.C. (2024). Betahistine in the Treatment of Peripheral Vertigo: An Evidence-Based Review. Egypt. J. Otolaryngol..

[94] Sanchez-Vanegas G., Castro-Moreno C.A., Buitrago D. (2020). Betahistine in the Treatment of Peripheral Vestibular Vertigo: Results of a Real-Life Study in Primary Care. Ear Nose Throat J..

[95] Parfenov V.A., Zamergrad M.V., Kazei D.V., Nauta J. (2020). A Study of the Efficacy and Safety of a New Modified-Release Betahistine Formulation in the Treatment of Vestibular Vertigo and Meniere’s Disease. S.S. Korsakov J. Neurol. Psychiatry.

[96] Bajenaru O., Roceanu A., Albu S., Zainea V., Pascu A., Georgescu M., Cozma S., Marceanu L.G., Muresanu D.F. (2014). Effects and Tolerability of Betahistine in Patients with Vestibular Vertigo: Results from the Romanian Contingent of the OSVaLD Study. Int. J. Gen. Med..

[97] Kabaya K., Tamai H., Okajima A., Minakata T., Kondo M., Nakayama M., Iwasaki S. (2022). Presence of Exacerbating Factors of Persistent Perceptual-postural Dizziness in Patients with Vestibular Symptoms at Initial Presentation. Laryngoscope Investig. Otolaryngol..

